# The Impact of Repeating Endosonography with Confocal Endomicroscopy for the Diagnosis of Cystic Neuroendocrine Tumor

**DOI:** 10.1155/2019/5187874

**Published:** 2019-01-14

**Authors:** Iqra Haq, Somashekar G. Krishna, Bhaveshkumar Patel, Thavam Thambi-Pillai, Chencheng Xie, Karah Odegaard, Kimberlee Buohy, Muslim Atiq

**Affiliations:** ^1^Department of Internal Medicine, University of South Dakota Sanford School of Medicine, Sioux Falls, South Dakota, USA; ^2^Division of Gastroenterology, Hepatology and Nutrition, The Ohio State University Wexner Medical Center, Columbus, Ohio, USA; ^3^Department of Gastroenterology, University of South Dakota Sanford School of Medicine, Sioux Falls, South Dakota, USA; ^4^Department of Surgery, University of South Dakota Sanford School of Medicine, Sioux Falls, South Dakota, USA; ^5^Department of Pathology, University of South Dakota Sanford School of Medicine, Sioux Falls, South Dakota, USA

## Abstract

Cystic pancreatic neuroendocrine tumors represent around 13% of all neuroendocrine tumors (Hurtado-Pardo 2017). There has been an increase in the incidence of cases due to improvement in imaging modalities. This is a case of a 68-year-old male with the incidental finding of a pancreatic cyst on CT. Initial Endoscopic Ultrasound with Fine Needle Aspiration (EUS-FNA) showed sonographic and cytology features suggestive of a pancreatic pseudocyst. However the cyst persisted with no change in size after aspiration leading to a follow-up EUS- FNA, which was combined with needle based confocal laser endomicroscopy (nCLE). The nCLE features were consistent with a cystic pancreatic neuroendocrine tumor, which was later confirmed on histology after surgical resection.

## 1. Introduction

Cystic tumors of the pancreas are being increasingly recognized as incidental findings with the advancement of imaging modalities. With reported prevalence being up to 13.5% in some studies [1, 2]. Management of indeterminate cystic lesions of the pancreas can be challenging, particularly with concern for early malignant cystic lesions of the pancreas. We are presenting a case in which nCLE was effectively utilized to make a definitive diagnosis.

## 2. Case Report

**Figure 1:**
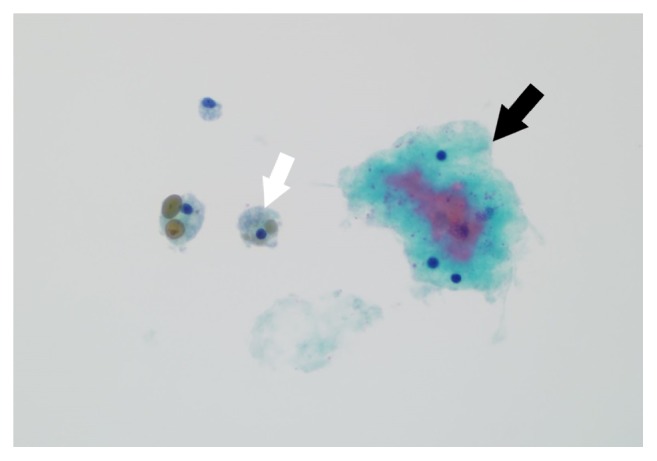
FNA cytology showing benign mesothelial cells (white arrow) and hemosiderin-laden macrophages (black arrow).

Our patient is a 68-year-old male with a past medical history of hyperlipidemia, hypertension, and smoking, who presented with an incidental pancreatic cyst on lung cancer screening helical CT. His CT had shown a 23 × 18 mm fluid density lesion in the distal pancreatic body, without pancreatic ductal dilation. He underwent an EUS-FNA which revealed an anechoic and septated cyst. Needle aspiration with a 19 G Boston Sci. needle was performed for amylase, tumor marker (CEA), and cytology. Cyst fluid analysis showed amylase of 1532 and a CEA of less than 200. FNA cytology revealed a moderately cellular aspirate with no identifiable malignant cells (Figure 1). These findings were consistent with a pseudocyst or a benign cyst.

**Figure 2:**
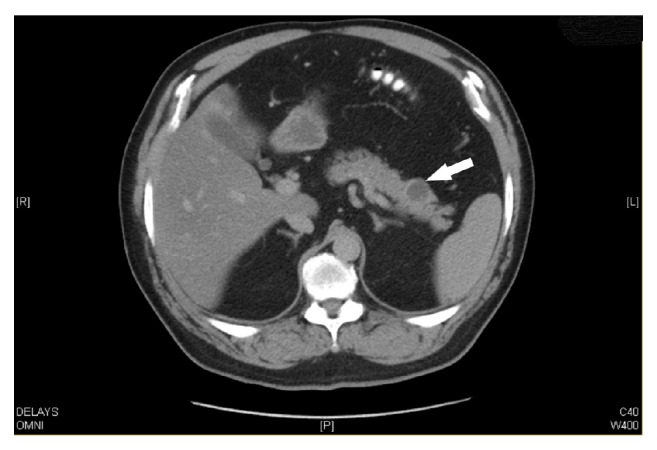
CT abdomen pelvis performed after EUS-FNA aspiration showing persistence of the cyst (arrow).

On follow-up CT abdomen and pelvis with IV contrast in six months, the cyst persisted and the size was unchanged (Figure 2).

**Figure 3:**
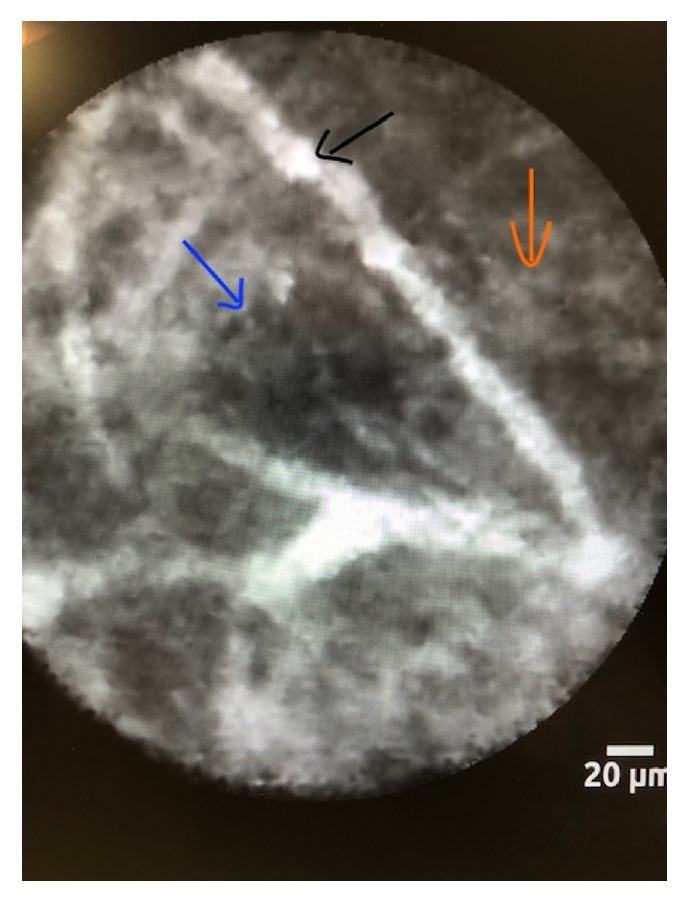
EUS guided needle based confocal laser endomicroscopy reveals clusters of cells (blue arrow) with surrounding areas of fibrosis (orange arrow) and vascularity (black arrow).

**Figure 4:**
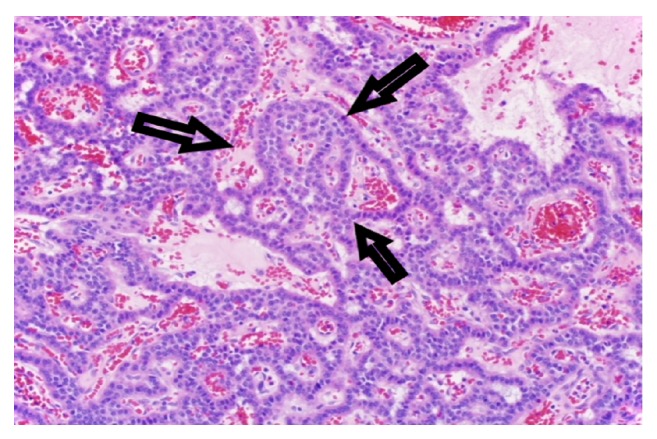
Histology showed a mass composed of trabeculae and solid nests of round, monotonous cells with crowded nuclei and stippled, open chromatin consistent with a cystic well-differentiated neuroendocrine tumor. Few mitotic figures were identified within the tumor.

This prompted a repeat EUS-FNA using 19G Boston Scientific needle combined with nCLE (using AQ-Flex 19; Mauna Kea Technologies). The tip of the AQ-Flex probe was advanced with the needle under EUS guidance until there was contact with the cyst wall without putting pressure. Fluorescein (2.5 to 5 mL of 10% Fluorescein) was injected intravenously immediately prior to CLE imaging. Around-3-minute-long video was acquired with permissible needle angulation. nCLE revealed thick cord like and dark nest like structures (Figures 3 and 4).

**Figure 5:**
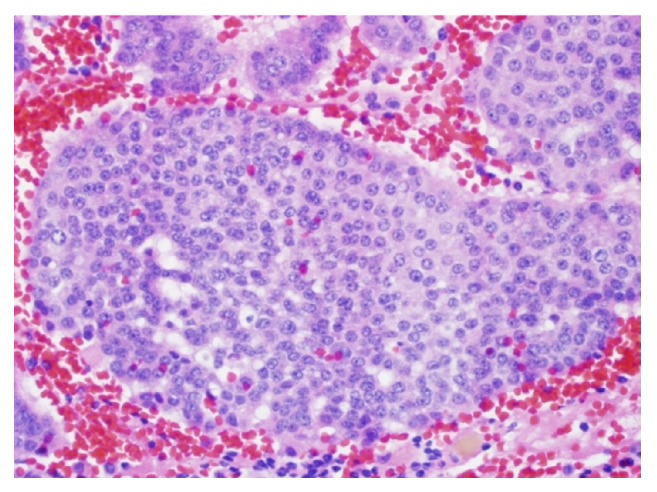
Histology showed a mass composed of trabeculae and solid nests of round, monotonous cells with crowded nuclei and stippled, open chromatin consistent with a cystic well-differentiated neuroendocrine tumor. Few mitotic figures were identified within the tumor.

There was no evidence for dark rings, vasculature network, or papillary projections to suggest intraductal papillary mucinous neoplasm. These findings were consistent with cystic neuroendocrine tumor of the pancreas [3]. These findings prompted us to send the patient for surgical evaluation. Final histopathology (Figures 4 and 5) confirmed the preoperative nCLE based diagnosis of the cystic neuroendocrine tumor of the pancreas.

## 3. Discussion

The differential diagnosis of cystic lesions of the pancreas includes pseudocysts, intraductal papillary mucinous neoplasms, mucinous cyst neoplasms, and serous cystadenoma.

These cystic tumors have a wide range of presentations on nCLE: intraductal papillary mucinous neoplasm, in which papillae can be visualized. Serous cystadenomas found to have a branching and tortuous network of multiple blood vessels in a “fern like” pattern, which has been termed as “superficial vascular network”; pseudocyst in which “clusters of bright, floating particles are found in a background of nondescript appearance lacking blood vessels”; finally, mucinous cystic neoplasms which present as solitary epithelial bands without papillae [2]. The nCLE findings in our case reveal nests and clusters of cells separated by stroma of the cyst. This pattern is diagnostic of a neuroendocrine tumor.

Incidences of pancreatic neuroendocrine tumors are rare and account for 1 to 2% of all pancreatic tumors [4]. Cystic neuroendocrine tumors of the pancreas are even rarer, comprising up to 3-17% of all the pancreatic neuroendocrine tumors [5]. Conventional imaging (CT scan and/or MRI with pancreatic protocol) has limited value in definitive diagnosis of cystic neuroendocrine tumors of the pancreas. EUS with FNA is a helpful tool in making the diagnosis. However, it does have its limitations which include small sample sizes and limited cells in the aspirated fluid which can decrease sensitivity.

In our case, EUS-FNA did not yield a correct diagnosis and the cyst persisted on repeat imaging at six-month follow-up. Therefore a nCLE was performed with repeat EUS-FNA, which revealed findings consistent with cystic neuroendocrine tumor of the pancreas. Recent studies have suggested that nCLE has shown promise in aiding diagnosis of solid tumors with good accuracy when compared to post-op diagnoses [6, 7]. There is evidence suggesting the use of nCLE in the work-up of cystic lesions of pancreas [3, 8–10].

Typically when evaluating cystic lesions greater than 2 to 3 cm of the pancreas, EUS with FNA is performed [11, 12]. This has proven to be superior to conventional imaging by most literature, but it can be very operator dependant. EUS with FNA has sensitivity and specificity at 91 and 94%, respectively. However, meta-analyses have shown that EUS-FNA has positive predictive value of 98% but a negative predictive value of 72% [13].

There are several studies on using nCLE for diagnosis of pancreatic masses, solid or cystic, which have shown promising results with an accuracy of ≥90% with low to no interobserver variability [6, 7, 14]. Krishna et al. concluded that nCLE is a good adjunct to use in an inconclusive EUS-FNA as in our case to differentiate mucinous versus nonmucinous primary cystic lesions (PCLs) as nCLE provides virtual histology of PCLs with a higher degree of accuracy, positive predictive value, and negative predictive value [15, 16].

The current IAP, AGA, and ACG guidelines indicate the use of EUS-FNA for evaluation of cysts >3 cm with no high-risk features or any size cyst with high-risk features; however the guidelines are not specific on indication and utility of nCLE in preoperative diagnoses as evidence is limited when compared to the gold standard of histopathology [12, 17, 18]. Currently recent European guidelines state that there is grade 1C level of evidence recommending against the use of nCLE for diagnosis of pancreatic cystic lesions [19]; however the same study suggests that this modality could be useful in preventing unnecessary surgical intervention in a selected number of patients.

Even though there are many studies to substantiate nCLE, it remains a modality that may be underutilized until it can be compared to gold standard (histopathology) in a large multicenter study.

## References

[1] de Jong K., Nio C. Y., Hermans J. J., Dijkgraaf M. G., Gouma D. J. (2010). High prevalence of pancreatic cysts detected by screening magnetic resonance imaging examinations. *Clinical Gastroenterology and Hepatology: the official clinical practice journal of the American Gastroenterological Association*.

[2] Lee K. S., Sekhar A., Rofsky N. M., Pedrosa I. (2010). Prevalence of incidental pancreatic cysts in the adult population on MR imaging. *American Journal of Gastroenterology*.

[3] Tsujino T., Huang J. Y., Samarasena J. B., Lemaistre A., Napoleon B., Chang K. J. (2016). EUS-Guided Needle-Based Confocal Laser Endomicroscopy in the Diagnosis of Cystic Pancreatic Neuroendocrine Tumor: Surgical Pathologic Correlation in Six Cases. *Gastrointestinal Endoscopy*.

[4] Hallet J., Law C. H. L., Cukier M., Saskin R., Liu N., Singh S. (2015). Exploring the rising incidence of neuroendocrine tumors: a population-based analysis of epidemiology, metastatic presentation, and outcomes. *Cancer*.

[5] Bordeianou L., Vagefi P. A., Sahani D. (2008). Cystic Pancreatic Endocrine Neoplasms: A Distinct Tumor Type?. *Journal of the American College of Surgeons*.

[6] Kongkam P., Pittayanon R., Sampatanukul P. (2016). Endoscopic ultrasound-guided needle-based confocal laser endomicroscopy for diagnosis of solid pancreatic lesions (ENES): a pilot study. *Endoscopy International Open*.

[7] Krishna S. G., Brugge W. R., Dewitt J. M. (2017). Needle-based confocal laser endomicroscopy for the diagnosis of pancreatic cystic lesions: an international external interobserver and intraobserver study (with videos). *Gastrointestinal Endoscopy*.

[8] Otaki F., Kedia P., Kumta N. A., Kahaleh M. (2015). Needle confocal microendoscopy of a pancreatic neuroendocrine tumor. *Gastrointestinal Endoscopy*.

[9] Kamboj A. K., Swanson B., Dillhoff M. E., Conwell D. L., Krishna S. G. (2017). Cystic pancreatic neuroendocrine tumors: correlation of in vivo needle-based confocal endomicroscopic findings by ex vivo analysis. *Gastrointestinal Endoscopy*.

[10] Krishna S. G., Lee J. H. (2016). Appraisal of needle-based confocal laser endomicroscopy in the diagnosis of pancreatic cysts. *World Journal of Gastroenterology*.

[11] Goh B. K. P., Ooi L. L. P. J., Tan Y. M. (2006). Clinico-pathological features of cystic pancreatic endocrine neoplasms and a comparison with their solid counterparts. *European Journal of Surgical Oncology*.

[12] Elta G. H., Enestvedt B. K., Sauer B. G. (2018). Diagnosis and Management of Pancreas cysts. *American Journal of Gastroenterology*.

[13] Hewitt M. J., McPhail M. J. W., Possamai L., Dhar A., Vlavianos P., Monahan K. J. (2012). EUS-guided FNA for diagnosis of solid pancreatic neoplasms: a meta-analysis. *Gastrointestinal Endoscopy*.

[14] Napoleon B., Lemaistre A.-I., Pujol B. (2016). In vivo characterization of pancreatic cystic lesions by needle-based confocal laser endomicroscopy (nCLE): proposition of a comprehensive nCLE classification confirmed by an external retrospective evaluation. *Surgical Endoscopy*.

[15] Giovannini M., Caillol F., Monges G. (2016). Endoscopic ultrasound-guided needle-based confocal laser endomicroscopy in solid pancreatic masses. *Endoscopy*.

[16] Konda V. J. A., Meining A., Jamil L. H. (2013). A pilot study of in vivo identification of pancreatic cystic neoplasms with needle-based confocal laser endomicroscopy under endosonographic guidance. *Endoscopy*.

[17] Tanaka M., Fernández-del Castillo C., Adsay V. (2012). International consensus guidelines 2012 for the management of IPMN and MCN of the pancreas. *Pancreatology*.

[18] Vege S. S., Ziring B., Jain R., Moayyedi P., Clinical Guidelines Committee (2015). American gastroenterological association institute guideline on the diagnosis and management of asymptomatic neoplastic pancreatic cysts. *Gastroenterology*.

[19] European Study Group on Cystic Tumours of the Pancreas (2018). European evidence-based guidelines on pancreatic cystic neoplasms. *Gut*.

